# A multiple technology-based physical activity intervention for Latina adolescents in the USA: randomized controlled trial study protocol for *Chicas Fuertes*

**DOI:** 10.1186/s13063-022-06105-2

**Published:** 2022-02-23

**Authors:** Britta Larsen, Emily Greenstadt, Brittany Olesen, Lilliana Osuna, Job Godino, Bess Marcus, Shira Dunsiger, Dawn Meyer, Michelle Zive

**Affiliations:** 1 Herbert Wertheim School of Public Health & Human Longevity Science, UC San Diego, La Jolla, CA USA; 2 Laura Rodriguez Research Institute, Family Health Centers of San Diego, San Diego, CA USA; 3 Department of Behavioral and Social Sciences, Brown University, Providence, RI USA; 4 Department of Neurosciences, School of Medicine, UC San Diego, La Jolla, CA USA

**Keywords:** Teens, mHealth, Health disparities, Exercise, Social media, Text messaging, Fitbit, Latinas, Randomized controlled trials

## Abstract

**Background:**

Latina adolescents in the USA report some of the lowest rates of physical activity of any demographic subgroup; this is paralleled by a markedly higher lifetime risk of obesity, type 2 diabetes, and other conditions related to inactivity. Despite this, to date, no fully powered clinical trials have tested physical activity interventions specifically for this population. High use of mobile technologies (including text messages, smartphone apps, and social media) suggests this could be an appropriate intervention channel, while also having potential for broad reach. This paper describes the protocol for *Chicas Fuertes*, a fully powered randomized trial of a mobile technology-based physical activity intervention for Latina adolescents.

**Methods:**

We plan to recruit 200 Latina teens (age 13–18) in San Diego, CA, currently engaging in ≤ 150 min/week of moderate-to-vigorous physical activity (MVPA) to be assigned 1:1 to the intervention or control groups. Those randomly assigned to the intervention group receive a one-on-one goal setting session followed by 6 months of mobile technology-based intervention, including a personalized website, Fitbit activity tracker and app, individually tailored text messages based on Fitbit data, and daily intervention content on Instagram. Those randomized to the control group receive only a Fitbit activity tracker. The main outcome is change in weekly minutes of MVPA from baseline to 6 months, measured both objectively (ActiGraph accelerometers and Fitbit Inspire HR) and subjectively (7-Day Physical Activity Recall Interview). Additional outcomes are maintenance of activity change at 12 months and changes in psychosocial constructs, including social support and self-efficacy, engagement with mobile technology channels, and costs of intervention delivery. We are also examining the potential mediators and moderators of the intervention. The efficacy of the intervention is analyzed using a mixed effects regression model, adjusting for any potential confounders not balanced by randomization. All analyses of accelerometer measured MVPA are also adjusted for wear time.

**Discussion:**

The *Chicas Fuertes* trial uses widely available mobile technologies to target critical health behavior, physical activity, in Latina teens, a population with a high lifetime risk of lifestyle-related diseases. The results will speak to the efficacy and acceptability of the intervention, which has the potential for broad dissemination.

**Trial registration:**

ClinicalTrials.govNCT04190225. Registered on November 20, 2019

## Introduction

### Background and rationale

Adolescent girls report the lowest levels of regular physical activity of any demographic group, particularly girls from racial/ethnic minority backgrounds [1–3]. While only 8% of adolescents in the USA meet the national guidelines of 60 min of moderate-to-vigorous physical activity (MVPA) per day [3], numbers are even worse when examined by gender and ethnicity, with only 2.9% of Mexican American adolescent girls meeting the guidelines (compared to 17.9% of Mexican American boys) [2]. These disparities in adolescents are paralleled by disparities in adulthood, with Latina women reporting less MVPA than with non-Latino White and non-Latino Black women and higher rates of lifestyle-related chronic disease, including overweight/obesity and diabetes [4–6]. Developing interventions to increase MVPA in Latina adolescents could improve not only their physical, psychosocial, and cognitive health during childhood [7], but also promote lifelong health habits that can reduce growing health disparities and promote health equity [8–10]. However, despite the low rates of MVPA in Latina adolescents and the implications of this for future health, to date, no large-scale trials of physical activity interventions specifically for Latina teens have been reported.

Low levels of MVPA in Latina teens may be partially due to psychosocial barriers in addition to environmental ones. Compared to adolescent boys, adolescent girls report lower self-efficacy, self-perception, social support, and perceived competence for physical activity, despite having equal access to facilities [11–16]. Latina teens have also reported lower support for activity than non-Latina White (White) girls, more negative support from boys, and less enjoyment for MVPA [17–19]. Successful MVPA interventions in this population thus will need to be grounded in theory and target psychosocial barriers experienced disproportionately by Latinas.

We conducted a single-arm pilot study of a web-based MVPA intervention for Latina teens (*N* = 21), adapted from an evidence-based web-delivered intervention for adult Latinas that was individually tailored based on social cognitive theory (SCT) [20] and the transtheoretical model [21, 22]. Participants engaged in a one-on-one coaching setting session to learn key behavior change techniques, such as goal setting, self-monitoring, and problem solving, and then filled out monthly questionnaires online to receive individually tailored MVPA information. The intervention was generally well-received, and participants increased their self-reported MVPA from 24.7(26.1) min/week to 79.4 (46.8) min/week over the 12-week intervention [23]. However, participants expressed a preference for more mobile technologies, shorter and more frequent intervention doses, and more visual intervention content.

To develop new intervention content and protocols, we conducted a series of iterative research, including interviews and focus groups with Latina teens (*N* = 50) [24]. Participants expressed a strong preference for an intervention designed and delivered exclusively for Latina girls, featuring images of realistic Latina girls with content in English but incorporating Spanish phrases. They also wanted content delivered via text messages and social media, with Instagram cited as the most popular platform, and expressed enthusiasm for other mobile technologies, such as wearable fitness trackers.

Incorporating these technologies into interventions has good potential for incorporating theory-based elements and would leverage rather than compete with the pervasive use of mobile technologies in teens. Given that 94% of teens use social media platforms [25], surprisingly few interventions have harnessed this tool to promote health behaviors in adolescents. One pilot study found that reminders and motivational messages posted to Instagram increased adherence to an MVPA program in female college students [26]. No large trials to date, however, have used Instagram as an intervention tool to increase MVPA. Relatedly, systematic reviews show good feasibility and acceptability for using text messaging to promote healthy lifestyles in teens, but only three studies used SMS to target activity and none with Latina girls [27–30].

There are also limited data on incorporating fitness trackers and smartwatches into interventions with teens, despite their growing popularity. Studies highlight that simply wearing a fitness tracker does not appear sufficient to increase activity [31]; however, trackers do appear to support increases in MVPA when used to reinforce more intensive online or in-person counseling [32, 33]. Again, however, there is limited evidence for this in adolescents and none in Latina girls.

### Objective

To respond to these gaps in the literature, we developed a multi-technology individually tailored theory-based MVPA intervention for Latina adolescents. The intervention incorporates a modified version of the website from the pilot trial, along with a coaching session, a wearable fitness tracker (Fitbit Inspire HR), individually tailored text messages, and Instagram posts. The aim of the current paper is to detail the study protocol for the randomized controlled trial testing the efficacy of this intervention.

## Methods: participants, intervention, and outcomes

### Trial design and setting

*Chicas Fuertes* is a parallel-group randomized trial. Adolescent Latinas (*N* = 200) living in San Diego County are randomized 1:1 to receive either the mobile technology-based MVPA intervention or only a wearable tracker (control group); analyses will test the superiority of the intervention over the control condition in increasing MVPA. Participants assigned to the intervention first receive a face-to-face counseling session to learn behavior change techniques. These sessions are conducted in a number of settings such as local parks, classrooms, and community-based organizations. They then receive a Fitbit and access to a tailored intervention website to reinforce goal setting and self-monitoring. Participants also are connected to a study Instagram account and receive regular text messages automatically generated by data from wearable trackers that update participants on goal progress and encourage adaptive goal setting. Activity is measured at baseline and follow-up via accelerometers and the 7-Day Physical Activity Recall (PAR) Interview and throughout the study using Fitbits. Measures are taken again at 12 months to evaluate the maintenance of activity gains. The sponsor and funder, the National Institutes of Health, played no part in study design. They have no role in the collection, management, analysis, and interpretation of the data. They played no part in the writing of the protocol and the decision to submit the protocol for publication. The study protocol uses the SPIRIT reporting guidelines [34].

### Participants

#### Eligibility criteria

Potential participants must self-identify as Latina; be 13–18 years old; read, write, and speak English: and be underactive, defined as regularly participating in MVPA for less than 150 min per week. Participants must also have regular (≥ 2 times/week) access to the Internet and to a cellphone that can receive and send text messages. Interested participants who do not have access to a smartphone are provided equipment to sync the Fitbit with a computer and use the Fitbit website dashboard rather than the smartphone app.

### Intervention

#### Intervention description

The intervention utilizes the same theory-based intervention strategies we have shown to successfully increase MVPA in non-Latino White men and women [35], Latina adults [36–38], Latino men [39], and which showed good potential efficacy with Latina adolescents in our pilot trial [23]. The current intervention focuses on the same core theoretical components and behavior change strategies from multiple psychosocial theories, including social cognitive theory (SCT) and the transtheoretical model (TTM), i.e., goal setting, self-monitoring, problem solving barriers, increasing social support, social norms, and rewarding oneself for meeting goals, which are reinforced by various technology channels: the Fitbit Inspire HR wrist monitor and Fitbit app, interactive automated text messaging, motivational content on social media (Instagram), and an interactive website. In order to leverage the tools of each channel and avoid fatigue with the technologies, different channels are emphasized at different time points. The schedule and content of the intervention components were refined through an intensive iterative process involving multiple interviews, focus groups, and design workshops with Latina girls (N = 50) and beta testing with a Youth Advisory Board; this has been described in detail elsewhere. Intervention components are summarized in Table 1.

**Table 1:** Intervention technology channels and content

Website content	Instagram posts	Fitbit tracker and smartphone App	SMS/texting
-New tailored report* -Goal setting calendar* -Current activity graphs* -Engagement points tally* -Activity resource guide -Activity leaders board -Message board -Monthly questionnaires -Instagram feed -Daily tip -Solutions to common barriers (*Individually tailored)	-Reminders to wear and sync Fitbits -Physical activity demonstration videos/modeling -Places to be active -Benefits/outcome expectancies -Self-efficacy -Social support -Behavior change techniques -Weekly challenge	-Goal setting (daily active minutes, steps, energy expenditure, standing) -Activity tracking -Reminders to move -Goal achievement reinforcement -Trophies and badges	-Weekly goal reminder -Weekly goal update* -Weekly adaptive goal setting* (*Months 2–12 only)

At the baseline one-on-one counseling session with a trained interventionist, the participant learns about MVPA (e.g. guidelines, benefits, intensity, duration) and engages in an individual goal setting. This session is based on principles of motivational interviewing, and teaches individuals to set realistic, specific short-term goals to build up to the long-term goal of meeting national guidelines (300 min/week of MVPA for children aged 12-17) [40]. The interventionist and the participant review her activity from the baseline week (measured by the Fitbit), and the participant selects her own goal for the following week. The interventionist also helps her identify personal barriers to activity (e.g., time, motivation), and teaches problem solving strategies. The participant is then oriented to the mHealth technology channels of the intervention. Health coaching sessions are recorded (with participant permission); a random subsample of recordings is evaluated by an external consultant to ensure fidelity of intervention delivery across participants.

#### Website

At the baseline study visit, participants assigned to the intervention group are provided with a unique login and password for the *Chicas Fuertes* website, which serves as a rich resource for MVPA tips and strategies, as well as the platform for MVPA goal setting and monitoring. The website includes a weekly calendar for planning out the upcoming week of activity, including which activities they plan to do, which days and times they plan to do them, where they plan to do them, and for how long. The calendar syncs with the participant’s Fitbit so that actual minutes of MVPA are directly imputed to allow participants to visualize planned vs. completed activity. The website also includes a monthly questionnaire, which measures participants’ attitudes, beliefs, and strategies for increasing MVPA. Responses to monthly questionnaires are fed into an expert system that automatically generates personalized reports, drawing from a bank of 330 messages addressing different levels of psychosocial and environmental factors affecting physical activity, such as stages of change, decisional balance, and self-efficacy, and tailors messages based on whether scores on these phenomena have increased, decreased, or remained stagnant. Tailored reports address (1) their current stage of motivational readiness for physical activity, (2) increasing self-efficacy for physical activity, (3) cognitive and behavioral strategies associated with physical activity behavior change (processes of change), (4) how the participant compares to their prior responses (progress feedback), (5) how the participant compares to other adolescents who are physically active (normative feedback), and (6) self-monitoring of MVPA (use of online activity logging calendars). Participants complete the questionnaire each month for the first 6 months of the study, and every other month for the remaining six months, and receive $10 each time they complete the questionnaire.

Other website features include maps of free or low-cost places to be active locally, strategies to overcome common barriers to MVPA, web links to free online workout videos, and a current activity leaders board that highlights the three most active participants that week (measured by Fitbit activity). The website also has an online message board, moderated by the interventionist, where participants can encourage each other to be active, congratulate and celebrate each other’s successes, and seek guidance or support. The website also scrolls the study Instagram feed (see below). To encourage regular use of the *Chicas Fuertes* website, participants receive “engagement points” for logging on, answering the weekly pop quiz question, and setting their weekly MVPA goals. Accrued points can be traded in for a variety of *Chicas Fuertes*-branded prizes, such as water bottles, power banks, and Bluetooth speakers.

#### Fitbit Inspire HR monitor and app

Participants in both the intervention and control groups are provided with a Fitbit Inspire HR monitor and free Fitbit app for the 1-year study; however, only those in the intervention group receive coaching on how to navigate the features of the Fitbit App. At baseline, the interventionist walks the intervention participant through the Fitbit app, focusing on how to monitor PA metrics, such as steps, MVPA minutes, and heart rate, as well as how to set goals for MVPA. Participants are encouraged to monitor their progress regularly on both the Fitbit wrist-worn tracker as well as the Fitbit app and to update their MVPA goals on the Fitbit app gradually over time as they work toward increasing their weekly MVPA minutes.

#### Text messaging

In the first month of the intervention, text messaging is primarily used to reinforce other intervention media channels. Participants receive text message reminders to use the website features, which include a link to take them directly to the website. They receive additional weekly texts reminding them to set goals and wear and sync their Fitbits. In months 2–12, participants also receive algorithm-derived individually tailored text messages updating them on their goal progress mid-week and reviewing MVPA at the end of the week. Data are sourced from the Fitabase software program that monitors Fitbit data in real time and computes total active and very active minutes. Texts encourage adaptive goal setting, asking participants who meet their activity minute goal if they can increase their goal by 10% for the next week until national guidelines are met. Participants who do not meet their goal receive a text calculating how many more minutes they need to add per day to meet their goal and asking if they believe that they can do it for the coming week.

#### Instagram posts

Instagram posts are used to deliver short, visual, daily intervention content based on specified constructs of psychosocial theories. At baseline, participants assigned to the intervention group are invited to “follow” the study’s private Instagram account. Focus group results prior to the study launch directed the development of Instagram posts that contain brightly colored images and videos featuring relatable girls engaging in MVPA, couched in positive and inspirational messaging. Each day of the week features one post based on constructs from the aforementioned psychosocial theories: benefits/outcomes, social support, modeling, environment, self-efficacy, and a weekly challenge incorporating behavior change techniques for participants to try during the week (see Fig. 3 for sample post). Along with daily posts, Instagram stories are posted throughout the week, which include interactive components such as a poll or quiz to engage users. Participants are encouraged to “like” the posts, which indicates to the study team that the post has been seen, and also ensures that *Chicas Fuertes* posts remain at the top of the participant’s Instagram feed.

#### One month call

After 1 month, participants receive a brief (20-min) phone call from the intervention staff to review progress. The interventionist reviews their goals and progress and helps them set new goals for the coming week and address barriers they have experienced so far. The participant is instructed to fill out their next online questionnaire to generate an individually tailored report on the website. Reminders to fill out questionnaires in subsequent months are automatically sent via text message.

#### Six-month visit

During the 6-month assessment, those in the intervention arm also engage in a repeat goal setting session and learn strategies to maximize MVPA maintenance over the next 6 months. The interventionist emphasizes continued self-monitoring using the Fitbit and asks them to identify sources of support to help them stay active.

#### Control group

The control group receives a Fitbit Inspire HR wrist-worn tracker to wear throughout the 12-month intervention. They are encouraged to wear the Fitbit every day and sync at least every 4 days. They have access to all features built into the Fitbit monitor and app (see Table 1) but do not receive explicit instruction on how to use or personalize them. This control group is meant to serve as a “real-world” control group, representing the large, growing population of individuals who purchase commercial fitness trackers and receive no additional guidance in using them.

#### Intervention: adherence

We examine adherence to the intervention through monitoring the frequency of Fitbit wear and syncing, tracking logins on the website, and monitoring views and likes on the Instagram account. Participants receive text messages reminding them to sync their Fitbit if they have not synced for 4 days. They also receive engagement points for interacting with the website, which can be traded in for prizes.

To increase enrollment and retention in the study, Fitbit devices and incentives in the form of cash payments and gift cards are provided to all participants. At baseline, each participant receives a Fitbit Inspire HR activity tracker to use throughout the study and keep after study participation is completed. Incentive payments of $25, $50, and $50 are provided at the baseline and 6- and 12-month follow-up measurement visits, respectively. Additionally, participants receive an additional $25 bonus if they complete all measures at the 6- and 12-month visits. Lastly, while in-person research was suspended at UC San Diego (March 2020 to August 2020) and is limited (August 2020 to present) to mitigate the spread of COVID-19, remote follow-up measurement visits are offered to participants unable to return in-person. This includes completing self-report surveys online.

### Outcomes

#### Primary outcome

The primary outcome is change in weekly minutes of MVPA from baseline to 6 months measured by the ActiGraph GT3X+ accelerometer. The ActiGraph GT3X+ is a hip-worn triaxial accelerometer which measures the movement and intensity of activity and has been validated against heart rate telemetry [41] and total energy expenditure [42], including in children [43]. Participants wear the ActiGraph for ≥ 12 h/day ≥ 7 days at baseline and follow-up. Participants receive regular text messages to remind them to wear the ActiGraph. Valid wear time is considered ≥ 3000 min on ≥ 4 days. Data is processed using Freedson’s age-specific cut points for children to identify activity at various intensities [3]. We will evaluate the total minutes of MVPA and total time in 10+ min bouts.

As an additional measure of MVPA change, at baseline and follow-up, participants also engage in the 7-Day PAR Interview. The 7-Day PAR is a semi-structured interview that assesses the frequency, duration, and intensity of MVPA. It has consistently demonstrated acceptable reliability, internal consistency, and congruent validity with objective MVPA measures [44, 45] and sensitivity to changes over time [46, 47]. It has been validated in children as young as 11 [48]. Administering the PAR requires annual certification. To ensure fidelity of PAR measures, a random sample of 5% of PAR interviews is recorded and assessed for fidelity by an external consultant.

#### Secondary outcomes and covariates

Participant demographic information is measured via a self-report questionnaire at baseline and includes age, race, and parents’ education, income, and marital status.

We will also assess the changes in trajectories of daily MVPA measured by the Fitbit Inspire HR. The Fitbit tracker measures physical activity intensity, energy expenditure, bouts of exercise, steps, distance traveled, and flights of stairs at varying resolutions. We will be evaluating the daily active minutes and steps recorded by the Fitbit.

We will also measure the technology engagement with the website, Fitbit, texting interactions, and Instagram. Engagement with the *Chicas Fuertes* website measures the number of logins and use of specific features, including goal setting, the weekly quiz, and monthly questionnaire completion. Fitbit use is measured as days worn and sync frequency, while texting is based on the percentage of interactive texts the participants responded to. Instagram engagement is measured by the number of views and likes by participants.

Questionnaires on the website are used to create individual intervention content and also serve as measures of psychosocial constructs. Stages of Change for Physical Activity (SCPA) is used to stage-match participants; it has been successful in previous trials and has acceptable reliability (kappa = 0.78; intraclass correlation *r* = 0.84) [49]. Processes of Change for Physical Activity (POC) was also administered monthly; POC contains 10 subscales on a variety of cognitive and behavioral processes related to MVPA change. Its subscale internal consistency ranges from .62 to .96 [50].. Self-Efficacy for physical activity (SE) was used to measure self-efficacy to become physically active across diverse contexts. The SE internal consistency is acceptable (alpha = .82) [49]. We also administered the Social Support for Exercise (SSE) scale which has three subscales of family, friends, and rewards and punishments and has acceptable internal consistency (alphas .61–.91) and criterion validity [51]. To assess enjoyment for physical activity, the Physical Activity Enjoyment Scale (PACES) is used to evaluate the level of personal satisfaction from PA. PACES has high internal consistency (alpha = 0.96) and test-retest reliability [52]. Lastly, we used the Outcome Expectations Scale to assess the beliefs regarding the consequences of physical activity participation will be examined by 9 items with internal consistency (alpha = .89) and validity based on confirmatory factor analysis and positive correlations with physical activity and self-efficacy.

Neighborhood environment is measured using the Neighborhood Environment Walkability Scale for Youth (NEWS-Y). The NEWS-Y measures perceptions of neighborhood environment, including recreation facility availability, pedestrian traffic safety, and walking facilities. It has shown acceptable reliability and correlations with objectively measured activity [53].

Participants also fill out the Children’s Depression Inventory at baseline and follow-up visits. The Children’s Depression Inventory (CDI) uses 27 self-report items to measure affective, cognitive, motivational, and somatic symptoms and has good reliability and validity [54].

Potential contamination is measured at 6 and 12 months through a contamination measure asking participants to indicate the number of people they know who were simultaneously in the study, how much contact they had with them, and if they changed any of their behaviors due to their contact with other participants.

At 12 months, participants in the intervention group were asked to complete a consumer satisfaction measure to assess the acceptability of the intervention. This is modified from our previous studies.

#### Participant timeline

**Fig. 1:**
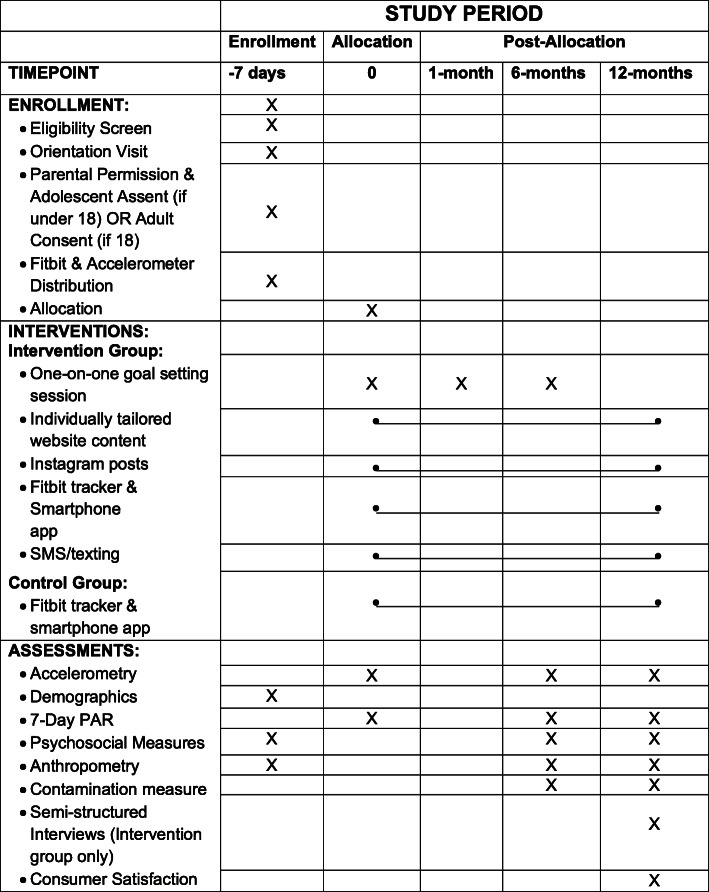
Schedule of enrollment, intervention, and assessment in the Chicas Fuertes study

Figure 1 provides an overview of the flow of participants through the study.

#### Screening and consenting

**Fig. 2:**
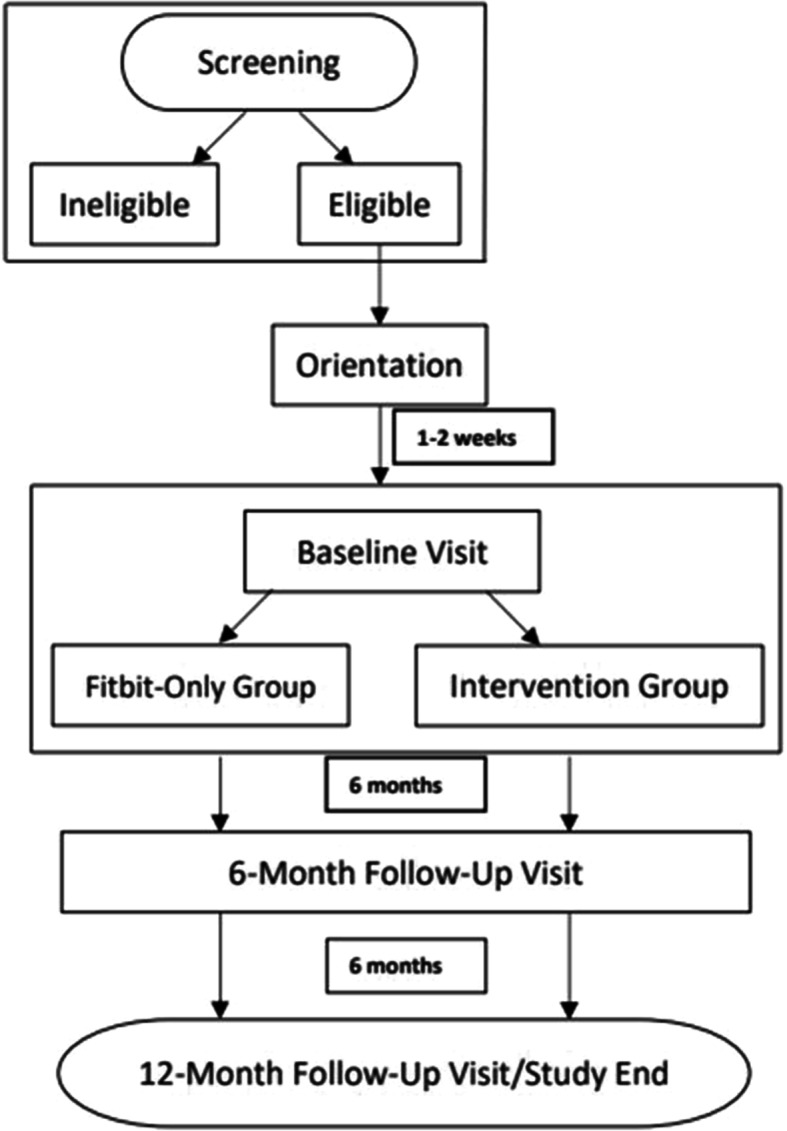
Study procedure flowchart

Potential participants may contact the research staff through Instagram direct message, Facebook, email, or text/call via the study phone number. Interested participants are then called to participate in the telephone screener that includes a description of the study purpose, procedures, risks, benefits, and eligibility requirements (Fig. 2). If the participant is under the age of 18, the research staff first talk with her parent/guardian before screening her to ask basic eligibility questions and to receive parental consent to screen the daughter, then speak to the adolescent to complete screening. If eligible, interested participants are invited to engage in an orientation session via Zoom.

#### Orientation visit

Prior to the orientation session, participants are instructed to download the Fitbit app and set up an account. They are also provided with an orientation packet via mail, including a Fitbit Inspire HR, a hip-worn accelerometer, and an orientation folder that includes a consent form, assent form, instructions on how to use the Fitbit, and instructions for wearing the accelerometer. The girl’s parent/guardian is invited to attend the first half of the orientation via Zoom to review a PowerPoint presentation that outlines the procedures, benefits, and expectations of the study. Following the presentations, the participant and their parent/guardian sign the written informed consent and assent forms, which are collected the following week at the baseline visit. The second half of the orientation is used to set up the Fitbit, including syncing it with a smartphone, connecting it to the study’s online Fitabase database, and temporarily blinding the Fitbit monitor and app to prevent feedback from influencing baseline activity. We also review the Fitbit and accelerometer expectations and schedule the baseline visit for 7 days after the orientation.

Over the next week, participants wear the Fitbit wrist monitor and a hip-worn GT3X+ accelerometer, with instructions to wear it 12 h per day for 7 days. Before the baseline visit, they also fill out online psychosocial measures, including self-efficacy, stage of change for activity, and social support (see Measures).

#### Baseline visit

One week after the orientation visit, participants attend an in-person baseline visit. Due to COVID-19 restrictions, in-person visits are conducted outdoors at parks close to the participant’s home. Participants return the accelerometer, which is assessed for sufficient wear time (≥ 3000 min on ≥ 4 days). After performing a short walk to demonstrate the activity of a moderate intensity level, participants complete the 7-Day PAR interview [44, 48]. Once all baseline measures are completed, including all online psychosocial questionnaires, participants are randomized to either the multi-media MVPA intervention or to a control group receiving only a Fitbit.

#### Follow-up visits

The primary outcome is change in MVPA from baseline to 6 months, with the maintenance of activity at 12 months serving as a secondary outcome. Follow-up visits are conducted remotely via the Zoom software. Prior to the 6- and 12-month visits, participants are mailed the accelerometer for 1 week of wear, similar to baseline. Accelerometers are mailed back and checked for wear time, after which participants complete the PAR interview over Zoom and fill in online psychosocial questionnaires. Participants also complete a contamination measure at 6 and 12 months to assess the amount and type of contact with participants in other conditions. At 12 months, a consumer satisfaction measure is completed by those in the Intervention arm to assess the acceptability of the Chicas Fuertes MVPA intervention, and a subset of participants engage in a one-on-one interview to give additional feedback about the program. Participants receive $50 for completing the baseline, 6- and 12-month measures, $25 for correctly wearing and returning the accelerometer, and $25 for completing all other measures. Participants receive an additional $25 bonus at month 12 if they complete all measures at all visits.

#### Sample size

Consistent with expert opinion [55, 56], we integrated several sources of evidence to determine an expected effect size for power calculations. First, based on our pilot findings [23], intervention participants increased their self-reported MVPA 24.7 (26.11) to 79.4 (11.3) at the end of treatment. Data from a recent study compared a Fitbit + texting intervention to Fitbit alone and found minimal increases in accelerometer-measured MVPA from baseline to end of treatment in their Fitbit-alone control group (32.7 (2.9) to 36.9 (3.4)) [31]. Thirdly, data from our previous study of a culturally and linguistically tailored web-based intervention among Latinas [37] showed significant increases in both self-reported and objectively (accelerometer) measured MVPA from baseline to 6 months for intervention versus web-based control. If we only consider the data from the youngest 50th percentile of participants, we see the effects sizes for between-group differences in self-reported MVPA from baseline to 6 months of *d* = 0.73 and *d* = 0.39 for objectively measured MVPA. Thus, we can consider three potential effect sizes for our power calculations: self-reported MVPA from pilot versus self-reported web-based control, self-reported web-based intervention arm versus self-reported web-based control, and objectively measured web-based intervention versus objectively measured Fitbit alone. Effect sizes were *d* = 0.68, *d* = 0.73, *d* = 0.73, respectively. Given these effect sizes and assuming a two-sided alpha = 0.05, we would need a total sample size of *N* = 126 to have 80% power to test intervention effects on both primary MVPA outcomes. However, given the potential risk for contamination from recruiting from schools (for example), and the known risks of powering on pilot studies, we have chosen to conservatively inflate our sample size to *N* = 200. Given a sample size of *N* = 200 (100/arm), we are more than adequately powered to detect differences > *d* = 0.40, which given our past studies and the state of the literature is more than reasonable.

#### Recruitment

**Fig. 3:**
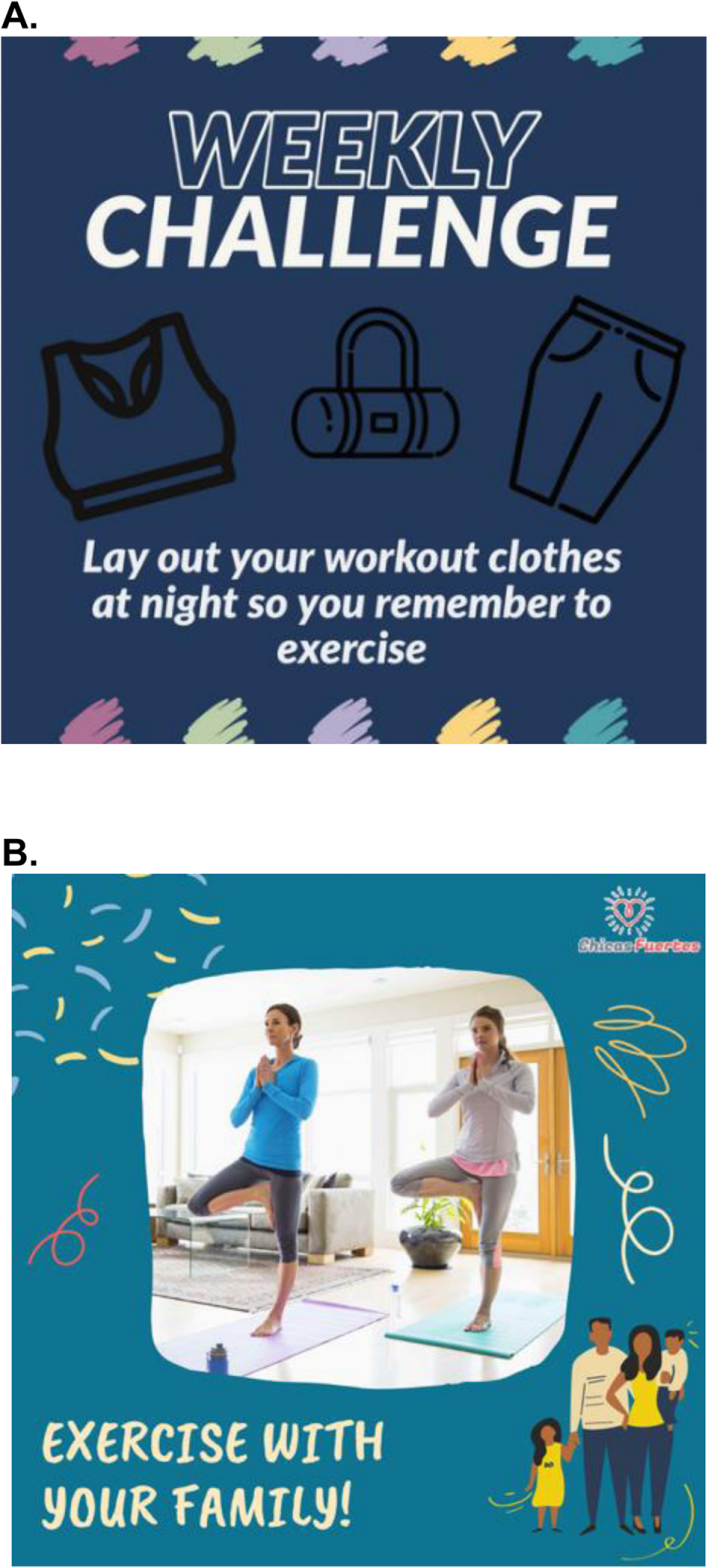
Sample Instagram posts showing (**a**) a weekly challenge and (**b**) social support

Over 3 years, we are recruiting a total of 200 adolescent girls (age 13–18) who self-identify as Latina and/or belong to groups considered Latino by the US Census Bureau. Recruitment is done through a variety of approaches including social media advertisements, providing health presentations in classes at local middle and high schools in San Diego County, conducting information sessions at community organization sites, distributing flyers through Latino-serving organizations, and contacting Latina women who have participated in previous studies and given permission to be contacted again to ask if they have family or friends who may be interested in participating (Fig. 3).

### Randomization, allocation concealment, and blinding

Randomization is performed using a random number sequence (generated based on a stratified block randomization procedure) programmed into the REDCap clinical trials software. Randomization is stratified by baseline stage of change according to the TTM (pre-contemplation, contemplation, or preparation) to ensure equal motivational readiness for MVPA adoption across groups. Study group assignment is placed in a sealed envelope based on an order determined by the randomization sequence, with different envelopes designated for each baseline stage of change. Randomization is performed by an interventionist who is not blinded to the participant study condition, while the staff members completing baseline and follow-up measures remain blinded to the condition. Siblings or close friends who enroll together are yoked together into a study arm to minimize contamination between arms; yoked groups are stratified across conditions to ensure equal distribution. After being randomized, participants’ Fitbit monitors and app are unblinded to allow for normal use. Participants in the intervention group then undergo a goal-setting session; those in the control group receive no more direct staff contact until the 6-month follow-up visit.

### Data management and quality assurance

#### Data management

The principal investigator will be responsible for monitoring the data collection, data quality and timeliness, and participant recruitment, accrual, and retention. All measures are collected and managed using REDCap, a secure, HIPAA-compliant web-based tool hosted at UCSD. REDCap provides an interface for data entry, audit trails for tracking data manipulation and export procedures, automated export procedures for data downloads to a variety of statistical software, and processes for importing data from external sources (e.g., body measurements and blood pressure).

#### Confidentiality

Data collected will be kept strictly confidential, accessed only by members of the trial team, and stored on a secure database on REDCap. Each participant will be allocated an individual trial identification number.

#### Statistical methods for primary and secondary outcomes

We will examine the effects of intervention vs. control on change in MVPA from baseline to 6 months (primary aim), measured both by accelerometers and the 7-Day PAR. To avoid the effect of outliers, we will apply a normalizing transformation (if necessary) to the outcome (*Y*_*i*,*j*_ = MVPA for participant *i* at follow-up *j*) prior to analysis. Using a series of mixed effects regression models, we will regress MVPA at follow-up on baseline, group, time, group × time, and confounders identified in the preliminary step. Objectively measured MVPA models will additionally adjust for wear time. Models will include random intercepts to adjust for repeated measures within participant over time. As an additional step, we will explore potential clustering by recruitment site (school or youth group) and control for contamination (binary risk indicator). The analysis will be conducted on the intent-to-treat sample with all participants randomized included in the analysis. Mixed effects models use a likelihood-based approach to estimation and thus do not require any direct imputation of missing outcomes. A similar approach will be used to evaluate maintenance of MVPA gains at 12 months; group effects will be parametrized so that a single model can assess the effects of treatment group on physical activity adoption (changes from baseline to 6 months) and maintenance (changes from 6 to 12 months). Should the data (MVPA) be skewed and transformations toward normality not successful, we will use longitudinal quantile regression models (which model median change instead of the mean).

We will apply two statistical approaches to handling missing data and compare these to the effect estimates from the primary outcome analysis (which takes a likelihood-based approach to estimation but does not directly impute missing data). The first is inverse probability weighting with propensity scores. This is a two-step method: first, we will model the probability of missingness as a function of baseline covariates and previous outcomes (using logistic regression). The resulting predicted probabilities of dropout will serve as the propensity scores. Next, the inverse propensity scores serve as weights in our regression model of the primary outcomes (i.e., reported weekly minutes of PA). Provided the data is missing at random (MAR) or that the probability of missingness can be fully explained by observable data, this approach produces asymptotically unbiased estimates. To allow for the possibility that the MAR assumption may not hold, we also will use pattern mixture models. In this case, the distribution of the primary outcome is assumed to follow a mixture of two distributions: one for those who complete follow-up and another for those who do not. This method allows us to quantify the robustness of the study findings to a range of missing data assumptions.

#### Statistics: additional analyses

We will explore the potential mediators of the intervention effect (e.g., self-efficacy, engagement, social support) using a multiple mediation approach, in which all potential mediators are tested simultaneously, using a product of coefficients method with bootstrapped standard errors (5000 samples with replacement). We will estimate the path coefficients (*a* path: effects of intervention on the changes in mediators from baseline to three and 5 months and *b* path: effects of changes in the mediators from baseline to three and 5 months on MVPA at 6-month follow-up, controlling for baseline), as well as the indirect effect of intervention (*ab* path: effect of intervention on MVPA through the mediators). Interest is in estimating the path coefficients, effect sizes, and confidence intervals, rather than strict hypothesis testing.

Using a similar analytic approach to that described for our primary aims, we will examine the potential moderators of the intervention effect on both MVPA outcomes, including age and neighborhood environment. Models will include themain effects of group, moderator, and group × moderator. A variable will be considered a moderator of the intervention effect if the interaction term is significantly different than zero.

Costs will be calculated from a payer perspective and will include all elements needed to deliver the intervention. A time tracking system will track staff time devoted to the intervention. Research activities unrelated to intervention delivery (e.g., obtaining consent) will also be tracked and removed from the analysis of intervention costs. Tracked costs will include materials (e.g., Fitbits), personnel time (for training and intervention delivery), and overhead (space, Internet, phone, etc.). We will also track the cost of modifying new technological features, such as the enhanced website and text messaging system, as well as the staff time needed to implement these features. Technology costs will be sourced directly from invoices from Illumina, Inc., which developed the website for the pilot study. Consistent with our previous studies, incremental cost-effectiveness ratios between conditions will be calculated as the differences in costs per minute increase in weekly physical activity [57, 58].

To examine patterns of daily MVPA and steps, we will model daily activity and steps from Fitbit using Gaussian process regression models [59]. Models will include separate long- and short-term trends for each of the arms along with common terms for weekly and monthly periodic trends and indicator variables for holidays. We will also use the time-varying effect model to examine how changes in these behaviors over time influence final MVPA [60].

### Oversight and monitoring

#### Roles and responsibilities: contributors, sponsor, funder, and committees

The funders have no role in the study design, data collection, analysis, decision to publish, or preparation of manuscripts. Authorship will follow the ICMJE guidelines.

A trial steering committee comprising the PI (Dr. Larsen), several co-investigators (Drs. Marcus, Godino, and Zive), and the study coordinator (Ms. Greenstadt) meets monthly to review conduct and progress of the trial. An operations committee comprising the PI (Dr. Larsen), the study coordinator (Ms. Greenstadt), and the Assessments Director (Ms. Olesen) meets weekly to review events and day to day operations. The study coordinator (Ms. Greenstadt) oversees all recruitment activities; consent and assent are gathered by trained research associates who have completed mandated ethics training for conducting human subjects research.

#### Data monitoring: formal committee, harms, and auditing

This is a single-site study that is considered low risk, and no planned interim analyses for efficacy or futility will be conducted. Therefore, a Data Safety and Monitoring Board is not appointed. However, adverse events and unanticipated problems involving risk to participants will be monitored continuously throughout the randomized controlled trial and reported to the Human Research Protections Programs (HRPP) at UC San Diego within 10 days. Anticipated adverse events include muscle or bone injury during physical activity and physical discomfort wearing the Fitbit device. There is no anticipated long-term harm for trial participation; thus, no provisions for post-trial care have been made. In the entirety of the study, the health and safety of participants will be held at the highest priority among the research team. The investigators shall inform participants of information relevant to their continued participation and pursue the research objectives with scientific diligence. The research staff, including the PI and study coordinator, meet weekly to review trial progress and events and discuss potential adverse events and necessary protocol amendments. Conduct is reviewed annually at UC San Diego through the HRPP. Protocol amendments will be submitted to the HRPP and approved, and will also be reported to the study sponsor and funder (NIH) and updated on the clinical trials registry. A protocol deviation form will be used to document any protocol deviations that occur.

#### Dissemination plans

After the completion of study analyses, or upon publication of findings, data will be made available to the scientific research community via a public website and/or data repository. Any data required to support the protocol can be supplied upon request. The results will be submitted for publication in scientific journals, and all participants will receive a summary of the findings.

## Discussion

The *Chicas Fuertes* study aims to test the efficacy of a multi-technology physical activity intervention for Latina adolescents. The main outcomes are change in minutes of weekly MVPA, along with daily MVPA measured by Fitbits, and changes in psychosocial constructs (e.g. self-efficacy, social support, depression symptoms). We will evaluate the use of different technology channels and dose effects on the main outcome, and evaluate potential moderators and mediators of the intervention to clarify how the intervention works and for whom.

Few previous physical activity interventions have targeted adolescent girls, and fewer still have targeted racial/ethnic minority adolescent girls, despite this group reporting the lowest levels of regular physical activity of any demographic group [1, 2, 61]. This study will utilize media channels that have not been used in previous large-scale trials with teens, such as Instagram and Fitbits, along with evidence-based channels such as web and texting [28, 62, 63]. By meeting teens in the digital space they already frequent, we will increase acceptability, maximize reach, and lower costs. Importantly, intervention content on these channels will support evidence-based theoretical constructs and behavior change techniques, such as goal setting, self-monitoring, self-efficacy, social support, and reinforcement [64, 65].

This study has a number of strengths. It targets a high-risk, underserved population, and was informed by thorough formative research, including a pilot trial, focus groups, and a youth advisory board of end-users that helped develop and refine intervention content and protocols [24]. It utilizes broad reach, low-cost intervention channels, maximizing the potential for dissemination, and will also evaluate cost-effectiveness and projected costs of implementation. It will use multiple validated measures of physical activity, including accelerometers, Fitbits, and the PAR interview, providing a rich longitudinal dataset of MVPA change.

There are also potential limitations to the current study, including potential contamination across study arms by recruiting participants from the same schools. This will be minimized by yoking family members into the same study condition and measuring and adjusting for potential contamination. Also, the study does not target other behaviors associated with chronic disease, such as diet. However, we felt it was important to first establish the efficacy of the intervention in increasing MVPA before targeting additional behaviors.

## Conclusion

This study will address a critical gap in public health literature and practice in promoting preventive health behaviors among ethnic minority adolescent girls. The results will speak to whether a low-touch, remotely delivered, home-based mobile technology intervention can effectively increase MVPA in a population suffering from low levels of activity. The results could inform the development and implementation of broadly reaching interventions in the community, academic, and clinical settings and could thus play an important role in the effort to prevent disease and promote health equity in Latinas both during adolescence and throughout their lifetime.

## Trial status

This study is approved by the Human Research Protections Programs at UCSD (protocol #182070). Recruitment began in August 2019 and is expected to conclude in March 2023.

## Data Availability

The datasets analyzed during the current study are available from the corresponding author on reasonable request.

## References

[1] Wolf AM, Gortmaker SL, Cheung L, Gray HM, Herzog DB, Colditz GA (1993). Activity, inactivity, and obesity: racial, ethnic, and age differences among schoolgirls. Am J Public Health..

[2] Whitt-Glover MC, Taylor WC, Floyd MF, Yore MM, Yancey AK, Matthews CE (2009). Disparities in physical activity and sedentary behaviors among US children and adolescents: prevalence, correlates, and intervention implications. J Public Health Policy..

[3] Troiano RP, Berrigan D, Dodd KW, Mâsse LC, Tilert T, McDowell M (2008). Physical activity in the United States measured by accelerometer. Med Sci Sports Exerc..

[4] Blackwell DL, Lucas JW, Clarke TC. Summary health statistics for U.S. adults: national health interview survey, 2012. Vital Health Stat 10. 2014(260):1-161. PMID: 24819891. 24819891

[5] Ogden CL, Carroll MD, Kit BK. Flegal KM. Prevalence of obesity among adults: United States, 2011-2012. NCHS Data Brief. 2013(131):1–8. PMID: 24152742 24152742

[6] Narayan KM, Boyle JP, Thompson TJ, Sorensen SW, Williamson DF (2003). Lifetime risk for diabetes mellitus in the United States. Jama..

[7] Janssen I, Leblanc AG (2010). Systematic review of the health benefits of physical activity and fitness in school-aged children and youth. Int J Behav Nutr Phys Act..

[8] Barnekow-Bergkvist M, Hedberg G, Janlert U, Jansson E (2001). Adolescent determinants of cardiovascular risk factors in adult men and women. Scand J Public Health..

[9] Schiller JS, Lucas JW, Ward BW, Peregoy JA. Summary health statistics for U.S. adults: National Health Interview Survey, 2010. Vital Health Stat 10. 2012;(252):1–207. PMID: 22834228 22834228

[10] Sacker A, Cable N (2006). Do adolescent leisure-time physical activities foster health and well-being in adulthood? Evidence from two British birth cohorts. Eur J Public Health..

[11] McKenzie TL, Marshall SJ, Sallis JF, Conway TL (2000). Student activity levels, lesson context, and teacher behavior during middle school physical education. Res Q Exerc Sport..

[12] McKenzie TL, Marshall SJ, Sallis JF, Conway TL (2000). Leisure-time physical activity in school environments: an observational study using SOPLAY. Prev Med..

[13] Robbins LB, Pender NJ, Kazanis AS (2003). Barriers to physical activity perceived by adolescent girls. J Midwifery Women’s Health..

[14] Biddle SJH, Whitehead SH, O’Donovan TM, Nevill ME (2005). Correlates of participation in physical activity for adolescent girls: a systematic review of recent literature. J Phys Act Health..

[15] Sallis JF, Zakarian JM, Hovell MF, Hofstetter CR (1996). Ethnic, socioeconomic, and sex differences in physical activity among adolescents. J Clin Epidemiol..

[16] Taylor WC, Yancey AK, Leslie J, Murray NG, Cummings SS, Sharkey SA, Wert C, James J, Miles O, McCarthy WJ (1999). Physical activity among African American and Latino middle school girls: consistent beliefs, expectations, and experiences across two sites. Women Health..

[17] Kelly EB, Parra-Medina D, Pfeiffer KA, Dowda M, Conway TL, Webber LS, Jobe JB, Going S, Pate RR (2010). Correlates of physical activity in Black, Hispanic, and White middle school girls. J Phys Act Health..

[18] Grieser M, Neumark-Sztainer D, Saksvig BI, Lee J-S, Felton GM, Kubik MY (2008). Black, Hispanic, and White girls’ perceptions of environmental and social support and enjoyment of physical activity. J Sch Health..

[19] Grieser M, Vu MB, Bedimo-Rung AL, Neumark-Sztainer D, Moody J, Young DR (2006). Physical activity attitudes, preferences, and practices in African American, Hispanic, and Caucasian girls. Health Educ Behav.

[20] Bandura A (1986). Social foundations of thought and action: a social cognitive theory.

[21] Prochaska JO, DiClemente CC (1986). Toward a comprehensive model of change. Treating addictive behaviors: processes of change. Applied clinical psychology.

[22] Prochaska JO, DiClemente CC, Norcross JC (1992). In search of how people change. Applications to addictive behaviors. Am Psychol..

[23] Larsen B, Benitez T, Cano M, Dunsiger SS, Marcus BH, Mendoza-Vasconez A, Sallis JF, Zive M (2018). Web-based physical activity intervention for Latina adolescents: feasibility, acceptability, and potential efficacy of the Niñas Saludables Study. J Med Internet Res..

[24] Larsen B, Greenstadt ED, Olesen BL, Marcus BH, Godino J, Zive MM (2021). An mHealth physical activity intervention for Latina adolescents: iterative design of the Chicas Fuertes Study. JMIR Form Res..

[25] Anderson M, Jiang J. Pew Research Center, May 2018. Teens, Social Media & Technology; 2018.

[26] Al-Eisa E, Al-Rushud A, Alghadir A, Anwer S, Al-Harbi B, Al-Sughaier N (2016). Effect of motivation by “Instagram” on adherence to physical activity among female college students. Biomed Res Int..

[27] Stephens J, Allen J (2013). Mobile phone interventions to increase physical activity and reduce weight: a systematic review. J Cardiovasc Nurs.

[28] Badawy MS, Kuhns ML (2017). Texting and mobile phone app interventions for improving adherence to preventive behavior in adolescents: a systematic review. JMIR Mhealth Uhealth..

[29] Hieftje K, Edelman EJ, Camenga DR, Fiellin LE (2013). Electronic media-based health interventions promoting behavior change in youth: a systematic review. JAMA Pediatr..

[30] Militello LK, Kelly SA, Melnyk BM (2012). Systematic review of text-messaging interventions to promote healthy behaviors in pediatric and adolescent populations: implications for clinical practice and research. Worldviews Evid Based Nurs..

[31] Wang JB, Cadmus-Bertram LA, Natarajan L, White MM, Madanat H, Nichols JF, Ayala GX, Pierce JP (2015). Wearable sensor/device (Fitbit One) and SMS text-messaging prompts to increase physical activity in overweight and obese adults: a randomized controlled trial. Telemed J E Health..

[32] Lewis ZH, Lyons EJ, Jarvis JM, Baillargeon J (2015). Using an electronic activity monitor system as an intervention modality: a systematic review. BMC Public Health..

[33] Hurling R, Catt M, Boni M, Fairley B, Hurst T, Murray P, et al. Using internet and mobile phone technology to deliver an automated physical activity program: randomized controlled trial. J Med Internet Res. 2007;9(2) 10.2196/jmir.9.2.e7. 10.2196/jmir.9.2.e7 PMC1874722 17478409

[34] Chan A-W, Tetzlaff JM, Gøtzsche PC, Altman DG, Mann H, Berlin JA (2013). SPIRIT 2013 explanation and elaboration: guidance for protocols of clinical trials. BMJ Br Med J.

[35] Marcus BH, Napolitano MA, King AC, Lewis BA, Whiteley JA, Albrecht A, Parisi A, Bock B, Pinto B, Sciamanna C, Jakicic J, Papandonatos GD (2007). Telephone versus print delivery of an individualized motivationally tailored physical activity intervention: Project STRIDE. Health Psychol..

[36] Marcus BH, Dunsiger SI, Pekmezi DW, Larsen BA, Bock BC, Gans KM, Marquez B, Morrow KM, Tilkemeier P (2013). The Seamos Saludables study: a randomized controlled physical activity trial of Latinas. Am J Prev Med..

[37] Marcus BH, Hartman SJ, Larsen BA, Pekmezi D, Dunsiger SI, Linke S, Marquez B, Gans KM, Bock BC, Mendoza-Vasconez AS, Noble ML, Rojas C (2016). Pasos Hacia La Salud: a randomized controlled trial of an internet-delivered physical activity intervention for Latinas. Int J Behav Nutr Phys Act..

[38] Pekmezi DW, Neighbors CJ, Lee CS, Gans KM, Bock BC, Morrow KM, Marquez B, Dunsiger S, Marcus BH (2009). A culturally adapted physical activity intervention for Latinas: a randomized controlled trial. Am J Prev Med..

[39] Larsen B, Dunsiger S, Hartman S, Nodora J, Pekmezi D, Marquez B (2014). Activo: assessing the feasibility of designing and implementing a physical activity intervention for Latino men. Int J Mens Health..

[40] Piercy KL, Troiano RP, Ballard RM, Carlson SA, Fulton JE, Galuska DA, George SM, Olson RD (2018). The physical activity guidelines for Americans. JAMA..

[41] Janz KF (1994). Validation of the CSA accelerometer for assessing children’s physical activity. Med Sci Sports Exerc..

[42] Melanson EL, Freedson PS (1995). Validity of the computer science and applications, Inc. (CSA) activity monitor. Med Sci Sports Exerc..

[43] Trost SG, Pate RR, Freedson PS, Sallis JF, Taylor WC (2000). Using objective physical activity measures with youth: how many days of monitoring are needed?. Med Sci Sports Exerc..

[44] Pereira MA, FitzerGerald SJ, Gregg EW, Joswiak ML, Ryan WJ, Suminski RR, Utter AC, Zmuda JM (1997). A collection of physical activity questionnaires for health-related research. Med Sci Sports Exerc..

[45] Rauh MJ, Hovell MF, Hofstetter CR, Sallis JF, Gleghorn A (1992). Reliability and validity of self-reported physical activity in Latinos. Int J Epidemiol..

[46] Dunn AL, Garcia ME, Marcus BH, Kampert JB, Kohl HW, Blair SN (1998). Six-month physical activity and fitness changes in Project Active, a randomized trial. Med Sci Sports Exerc..

[47] Dunn AL, Marcus BH, Kampert JB, Garcia ME, Kohl HW, Blair SN (1999). Comparison of lifestyle and structured interventions to increase physical activity and cardiorespiratory fitness: a randomized trial. JAMA..

[48] Sallis JF, Buono MJ, Roby JJ, Micale FG, Nelson JA (1993). Seven-day recall and other physical activity self-reports in children and adolescents. Med Sci Sports Exerc..

[49] Marcus BH, Selby VC, Niaura RS, Rossi JS (1992). Self-efficacy and the stages of exercise behavior change. Res Q Exerc Sport..

[50] Marcus BH, Rossi JS, Selby VC, Niaura RS, Abrams DB (1992). The stages and processes of exercise adoption and maintenance in a worksite sample. Health Psychol.

[51] Sallis JF, Grossman RM, Pinski RB, Patterson TL, Nader PR (1987). The development of scales to measure social support for diet and exercise behaviors. Prev Med..

[52] Kendzierski D, DeCarlo K (1991). Physical Activity Enjoyment Scale: two validation studies. J Sport Exerc Psychol.

[53] Rosenberg D, Ding D, Sallis JF, Kerr J, Norman GJ, Durant N, Harris SK, Saelens BE (2009). Neighborhood Environment Walkability Scale for Youth (NEWS-Y): reliability and relationship with physical activity. Prev Med..

[54] Kovacs M (1992). The Children’s Depression Inventory (CDI) Manual.

[55] Leon AC, Davis LL, Kraemer HC (2011). The role and interpretation of pilot studies in clinical research. J Psychiatr Res..

[56] Kraemer HC, Mintz J, Noda A, Tinklenberg J, Yesavage JA (2006). Caution regarding the use of pilot studies to guide power calculations for study proposals. Arch Gen Psychiatry..

[57] Larsen B, Marcus B, Pekmezi D, Hartman S, Gilmer T (2017). A web-based physical activity intervention for Spanish-speaking Latinas: a costs and cost-effectiveness analysis. J Med Internet Res.

[58] Larsen B, Gilmer T, Pekmezi D, Napolitano MA, Marcus BH (2015). Cost effectiveness of a mail-delivered individually tailored physical activity intervention for Latinas vs. a mailed contact control. Int J Behav Nutr Phys Act.

[59] Gelman A, Carlin JB, Stern HS, Dunson DB, Vehtari A, Rubin DB (2014). Bayesian data analysis. Third edition. ed.

[60] Tan X, Shiyko MP, Li R, Li Y, Dierker L (2012). A time-varying effect model for intensive longitudinal data. Psychol Methods..

[61] Andersen RE, Crespo CJ, Bartlett SJ, Cheskin LJ, Pratt M (1998). Relationship of physical activity and television watching with body weight and level of fatness among children: results from the Third National Health and Nutrition Examination Survey. JAMA..

[62] Webb TL, Joseph J, Yardley L, Michie S (2010). Using the internet to promote health behavior change: a systematic review and meta-analysis of the impact of theoretical basis, use of behavior change techniques, and mode of delivery on efficacy. J Med Internet Res..

[63] Lau PW, Lau EY, Wong del P, Ransdell L. A systematic review of information and communication technology-based interventions for promoting physical activity behavior change in children and adolescents. J Med Internet Res. 2011;13(3):e48, 10.2196/jmir.1533. 10.2196/jmir.1533 PMC3222183 21749967

[64] Sallis JF, Prochaska JJ, Taylor WC (2000). A review of correlates of physical activity of children and adolescents. Med Sci Sports Exerc..

[65] van Sluijs EM, McMinn AM, Griffin SJ (2007). Effectiveness of interventions to promote physical activity in children and adolescents: systematic review of controlled trials. Bmj..

